# Excessive unilateral proliferation of spermatogonia in a patient with non-obstructive azoospermia – adverse effect of clomiphene citrate pre-treatment?

**DOI:** 10.1186/s12610-020-00111-7

**Published:** 2020-09-01

**Authors:** Daniela Fietz, Adrian Pilatz, Thorsten Diemer, Florian Wagenlehner, Martin Bergmann, Hans-Christian Schuppe

**Affiliations:** 1 Institute for Veterinary Anatomy, Histology and Embryology, Justus Liebig University Giessen, Frankfurter Straße 98, 35392 Giessen, Germany; 2 Hessian Centre of Reproductive Medicine, Justus Liebig University Giessen, 35392 Giessen, Germany; 3 Department of Urology, Pediatric Urology and Andrology, Justus Liebig University Giessen, 35392 Giessen, Germany

**Keywords:** Azoospermia, Clomiphene citrate, Male infertility, Spermatogonia, Blood-testis barrier, Sertoli cell function, Azoospermie, Citrate de Clomifène, Infertilité Masculine, Spermatogonies, Barrière hémato-testiculaire, Fonction de la Cellule de Sertoli

## Abstract

**Background:**

Clomiphene citrate has been proposed as pre-treatment for infertile men with non-obstructive, testicular azoospermia (NOA) before surgery for testicular sperm extraction (TESE), especially when serum testosterone is low.

**Case presentation:**

Here, we report on a 33-year old azoospermic patient with a previous history of repeated “fresh” TESE and clomiphene citrate therapy (50 mg/day over 6 months) before undergoing microscopically assisted, bilateral testicular biopsy. Comprehensive histological and immunohistochemical work-up revealed a heterogeneous spermatogenic arrest at the level of spermatogonia or primary spermatocytes, with focally preserved spermatogenesis up to elongated spermatids in the right testis. In the left testis, the majority of tubules (> 70%) showed no tubular lumen or regular seminiferous epithelium but a great number of spermatogonia-like cells. These cells proved to be normally differentiated spermatogonia (positive for melanoma associated antigen 4 (MAGEA4), negative for placental alkaline phosphatase (PlAP)) with increased proliferative activity (positive for proliferating cell nuclear antigen (PCNA)) and a slightly higher rate of apoptotic cells. When compared to a tissue control with normal spermatogenesis, expression of sex hormone receptors androgen receptor (AR), estrogen receptor (ER) alpha, and G-protein coupled estrogen receptor 1 (GPER1) was not altered in patient samples. Sertoli cells appeared to be mature (positive for vimentin, negative for cytokeratin 18), whereas the expression of zona occludens protein 1 (ZO-1), claudin 11, and connexin 43 was absent or dislocated in the tubules with abundance of spermatogonia.

**Conclusion:**

This result suggests that formation of the blood-testis barrier is disturbed in affected tubules. To our knowledge this is the first observation of excessive, non-malignant proliferation of spermatogonia in a NOA patient. Although underlying molecular mechanisms remain to be elucidated, we hypothesize that the unusual pathology was triggered by the high-dose clomiphene citrate treatment preceding testicular biopsy.

## Résumé

**Contexte:**

Chez les hommes infertiles qui présentent une azoospermie non obstructive (NOA), le citrate de clomifène a été proposé comme pré-traitement avant la chirurgie pour extraction testiculaire de spermatozoïdes (TESE), surtout quand la testostérone sérique est basse.

**Présentation du cas:**

Nous rendons compte, ici, d’un patient azoospermique de 33 ans avec antécédent de traitements répétés par TESE « frais » et par citrate de clomifène (50 mg/jour sur 6 mois) avant de subir une biopsie testiculaire bilatérale assistée microscopiquement.

L’étude histologique et immunohistochimique a révélé un arrêt hétérogène de la spermatogénèse au stade de spermatogonies ou de spermatocytes primaires, avec des foyers de spermatogenèse préservée jusqu’au stade de spermatides allongées dans le testicule droit.

Dans le testicule gauche, la majorité des tubules (>70%) ne présentaient ni lumière tubulaire ni épithélium séminifère régulier mais un grand nombre de cellules spermatogonies-like. Ces cellules se sont avérées être des spermatogonies normalement différenciées (positives pour l’antigène 4 associé au mélanome (MAGEA4), négatives pour la phosphatase alcaline placentaire (PlAP)) avec une activité proliférative accrue (positives pour l’antigène nucléaire de prolifération cellulaire (PCNA)) et un taux un peu plus élevé de cellules apoptotiques. Comparée à celle d’un tissu témoin avec spermatogenèse normale, l’expression des récepteurs aux hormones sexuelles, récepteur aux androgènes (AR), récepteur aux estrogènes (ER) alpha et récepteur 1 à la protéine G couplée aux estrogènes (GPER1), n’était pas modifiée dans les échantillons du patient. Les cellules de Sertoli semblaient matures (positives à la vimentine, négatives cytokératine 18), tandis que l’expression de la protéine 1 de la zone occludens (ZO-1), de la claudine 11, et de la connexine 43 était absente ou délocalisée dans les tubules présentant une abondance de spermatogonies.

**Conclusion:**

Ces résultats suggèrent que la formation de la barrière hémato-testiculaire est perturbée dans les tubules affectés. À notre connaissance, il s’agit de la première observation d’une prolifération excessive et non maligne de spermatogonies chez un patient avec NOA. Bien que les mécanismes moléculaires sous-jacents restent à élucider, nous supposons que cette pathologie inhabituelle a été déclenchée par le traitement au citrate de clomifène à haute dose précédant la biopsie testiculaire.

## Background

Male factor infertility is involved in approximately 50% of couples unable to conceive within 12 months of regular, unprotected intercourse [1]. Among those men referred for andrological diagnostic work-up, azoospermia is observed in 10–15% of cases, with the majority of patients suffering non-obstructive azoospermia (NOA) [2, 3]. In contrast to treatable forms of hypogonadotrophic hypogonadism, primary testicular failure resulting in NOA represents the most severe form of male factor infertility [4]. At the histological level, patterns of testicular damage range from hypospermatogenesis and spermatogenic arrest to Sertoli cell-only syndrome [for review see [5, 6]. Underlying etiologies are largely heterogeneous and include both congenital and acquired disorders, such as Klinefelter’s syndrome, cryptorchidism, orchitis, or toxic/iatrogenic insults [7]. In a significant proportion of patients no cause can be identified (idiopathic azoospermia). With the introduction of intracytoplasmic sperm injection (ICSI), surgical sperm retrieval became a therapeutic option. Open, bilateral biopsy has been recommended for testicular sperm extraction (TESE), which is successful in about 50% of NOA patients [7, 8]. Whether microdissection under an operating microscope (mTESE) is superior to the conventional procedure (cTESE) remains a matter of ongoing debate [8].

While definitive non-invasive markers predicting successful sperm retrieval for infertile men with NOA are lacking, hormonal pre-treatment in order to increase intratesticular testosterone levels has been proposed [9, 10]. Therapeutic concepts are similar to those used empirically in cases of idiopathic oligozoospermia and comprise anti-estrogens such as clomiphene citrate (CC) and tamoxifen [4, 11]. From their meta-analysis of available studies, Chua et al. [12] concluded that anti-estrogens have beneficial effects on sperm concentration and pregnancy rates when prescribed for idiopathic male infertility. In NOA patients treated with CC, sperm retrieval rates were significantly higher compared to untreated controls, and in some men return of sperm to the ejaculate was reported [9, 13]. Other studies, however, produced conflicting results [10]. Randomized controlled trials investigating anti-estrogens for improvement of TESE success are unavailable to date.

Here, we report on a 33-year old azoospermic patient with a previous history of repeated “fresh” TESE and off-label CC therapy over 6 months before undergoing combined multi-focal and microscopically assisted, bilateral testicular biopsy.

## Case presentation

### Patient history

The 33-year old patient presented with couple infertility. Initial andrological work-up in another center 5 years earlier had revealed azoospermia, without any clinical signs or symptoms. Hormone analyses were within normal range (Table 1), whereas genetic testing showed a heterozygous cystic fibrosis transmembrane conductance regulator (CFTR) gene mutation (R117H), but neither karyotype nor Y chromosome abnormalities. Available medical reports indicated a first bilateral TESE with cryopreservation of few testicular sperm of insufficient quality, followed by a treatment with 25 mg CC per day over 3 months in order to increase gonadotropin levels (Table 1). Subsequently, a second bilateral “fresh” TESE, allowed for successful in vitro fertilization (IVF)/ICSI, resulting in a pregnancy and birth of a healthy son. With the desire to have another child, the patient had a third bilateral “fresh” TESE 3 years later alio loco, after he had restarted CC therapy (50 mg/day, over 1.5 months). As sperm retrieval remained negative, he continued CC therapy (50 mg/day) and was referred to our center for repeated testicular surgery 6 months later. From previous procedures, neither histology, nor cryopreserved sperm were available.

**Table 1:** Hormonal follow-up of a patient with non-obstructive azoospermia before, during, and after clomiphene citrate treatment

	***09/2013***	***04/2014***^**a**^	***01/2018***^**a**^	***05/2018***^**a**^	***07/2018***^**b**^
**FSH** [IU/l] (1.0–10.0)	2.1	5.3	9.2	8.0	7.3
**LH** [IU/l] (1.0–9.0)	2.6	9.8	8.3	8.8	5.5
**Testosterone** [ng/ml] (3.50–10.00)	3.9	14.6	13.5	9.1	6.8
**Estradiol** [pg/ml] (11–41)	15.8	–	101	82	52
**SHBG** [nmol/l] (17.3–65.8)	28.2	–	43.0	40.0	41.5

^a^during clomiphene citrate treatment; ^b^after 4th testicular biopsy / TESE

*FSH* follicle stimulating hormone, *LH* luteinizing hormone, *SHBG* sex hormone binding globulin, *IU/l* international units per liter, ng/ml: nanogram per milliliter, pg/ml: picogram per milliliter, nmol/l: nanomole per liter

Clinical examination showed no genital abnormalities, with testis volumes of 20 ml (left) and 13 ml (right), as well as bilaterally palpable vasa deferentia. Ultrasound revealed regular organ patterns, i.e. normal testicular texture. Agenesis of seminal vesicles or kidney could be excluded. Semen analysis confirmed azoospermia, while signs of infection/inflammation or accessory gland dysfunction were not detected (Suppl. Table 1). Endocrine parameters and their longitudinal follow-up are compiled in Table 1. Testicular surgery was performed bilaterally under general anesthesia as described elsewhere (combined trifocal and microscopically assisted approach) [14]. Tissue specimens from each retrieval site were subjected to histological evaluation as well as cryopreservation (TESE).

### Histological evaluation of testicular biopsies

**Fig. 1:**
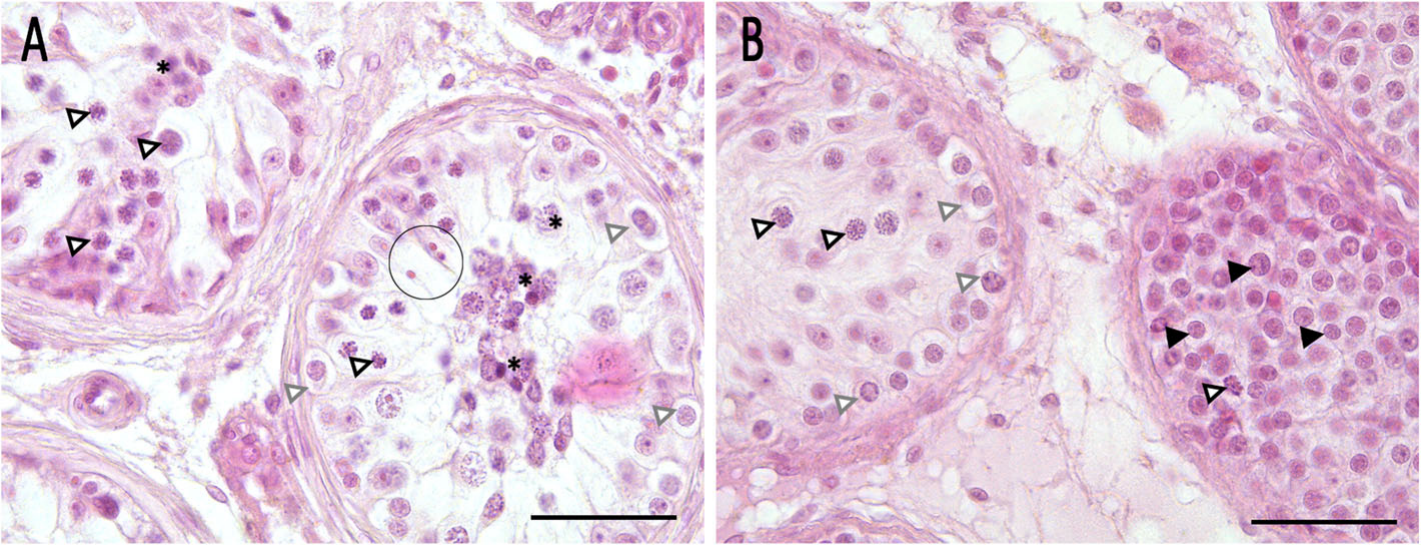
Histological evaluation. **a** Representative picture of the right testis, taken from the middle section. Two tubules are shown, the left one presenting only primary spermatocytes (lined arrowheads) and degenerating germ cells (asterisk), whereas the right tubule shows additionally a small group of elongated spermatids (circle). Gray lined arrowheads: spermatogonia. **b** Representative picture of the left testis, taken from the lower pole. Two tubules are shown, the left one presenting single primary spermatocytes (lined arrowheads) and spermatogonia (gray lined arrowheads) correctly aligned to the basal membrane, and the right tubule showing high numbers of spermatogonia/spermatogonia-like cells, dislocated and no longer restricted to the basal membrane (black arrowheads) with a single spermatocyte. Hematoxylin eosin (HE) staining, bar 50 μm

Processing of testicular tissue, histological evaluation and score count analysis of spermatogenesis were performed as previously described [6, 15] (see Suppl. Material). Score count analysis of all available biopsies revealed severely disrupted spermatogenesis (Suppl. Fig. 1). In the right testis, single seminiferous tubules with qualitatively preserved, but quantitatively severely reduced spermatogenesis (hypospermatogenesis) was detected in three of four biopsies, accompanied with a focal tubular atrophy and arrest of spermatogenesis at the level of primary spermatocytes (representative picture shown in Fig. 1a). Score counts ranged from 0 (no tubules with elongated spermatids) to 0.1 (1% of tubules with at least single elongated spermatids) (Fig. 1a). In the left testis, tubules showed either only spermatogonia (i.e. arrest of spermatogenesis at the level of spermatogonia, score count = 0) or spermatogonia and single primary spermatocytes (i.e. arrest of spermatogenesis at the level of primary spermatocytes, score count = 0) (summarized as “spermatogonial arrest” for short characterization of the leading pathology). Most interestingly, tubules showing a massive increase in numbers of spermatogonia-like cells were prevalent (representative picture shown in Fig. 1b). To compare the number of spermatogonia/spermatogonia-like cells in patient and control tissues, a semi-quantitative evaluation by counting spermatogonia in 25 randomly chosen tubules in each sample was performed (for details, see Suppl. Material, Suppl. Fig. 2). A biopsy obtained from a patient with obstructive azoospermia and normal spermatogenesis (NSP) served as control. By this approach, we were able to show a significantly higher number of cells in the tubules of the patient’s left testis packed with spermatogonia-like cells compared to NSP (Suppl. Fig. 2). In both testes, morphology of somatic Sertoli cells and Leydig cells appeared normal. By histological evaluation, no signs of malignancy were detected (immunohistochemistry addressing PlAP proved to be negative; not shown).

### Immunohistochemistry

**Fig. 2:**
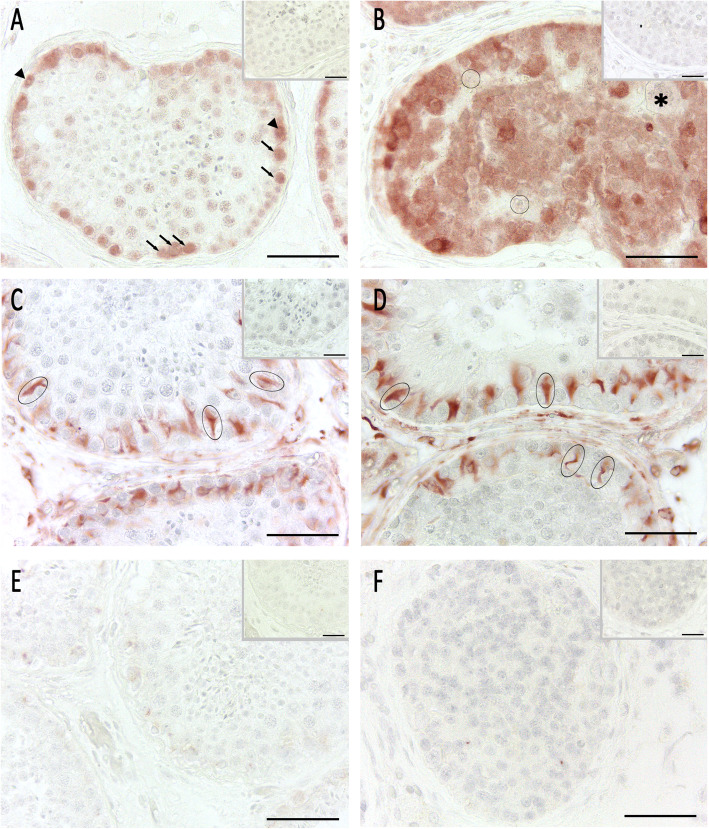
Immunohistochemical analysis of germ and Sertoli cells. Pictures **a**, **c**, and **e** show intact spermatogenesis (NSP) as control, pictures **b**, **d**, and **f** show spermatogonial arrest (patient’s left testis). **a**, **b** By using germ cell marker MAGEA4, we were able to differentiate A_dark_ spermatogonia (black arrowheads) and A_pale_ spermatogonia (black arrows) in NSP. In spermatogonial arrest, cells were MAGEA4-positive and therefore identified as spermatogonia. Sertoli cells (circles) and single megalospermatocytes (asterisk) were not stained. **c**, **d** Vimentin was present in Sertoli cell cytoplasm (ovals) in NSP and arrest. The upper tubule in D contains single spermatocytes and shows normal vimentin distribution. In the lower tubule, less intense vimentin staining in Sertoli cells were visible. **e**, **f** No specific cytokeratin 18 staining was detected in NSP and arrested spermatogenesis. Aminoethyl carbazole (AEC) detection, bar 50 μm, inset as negative control without first antibody

**Fig. 3:**
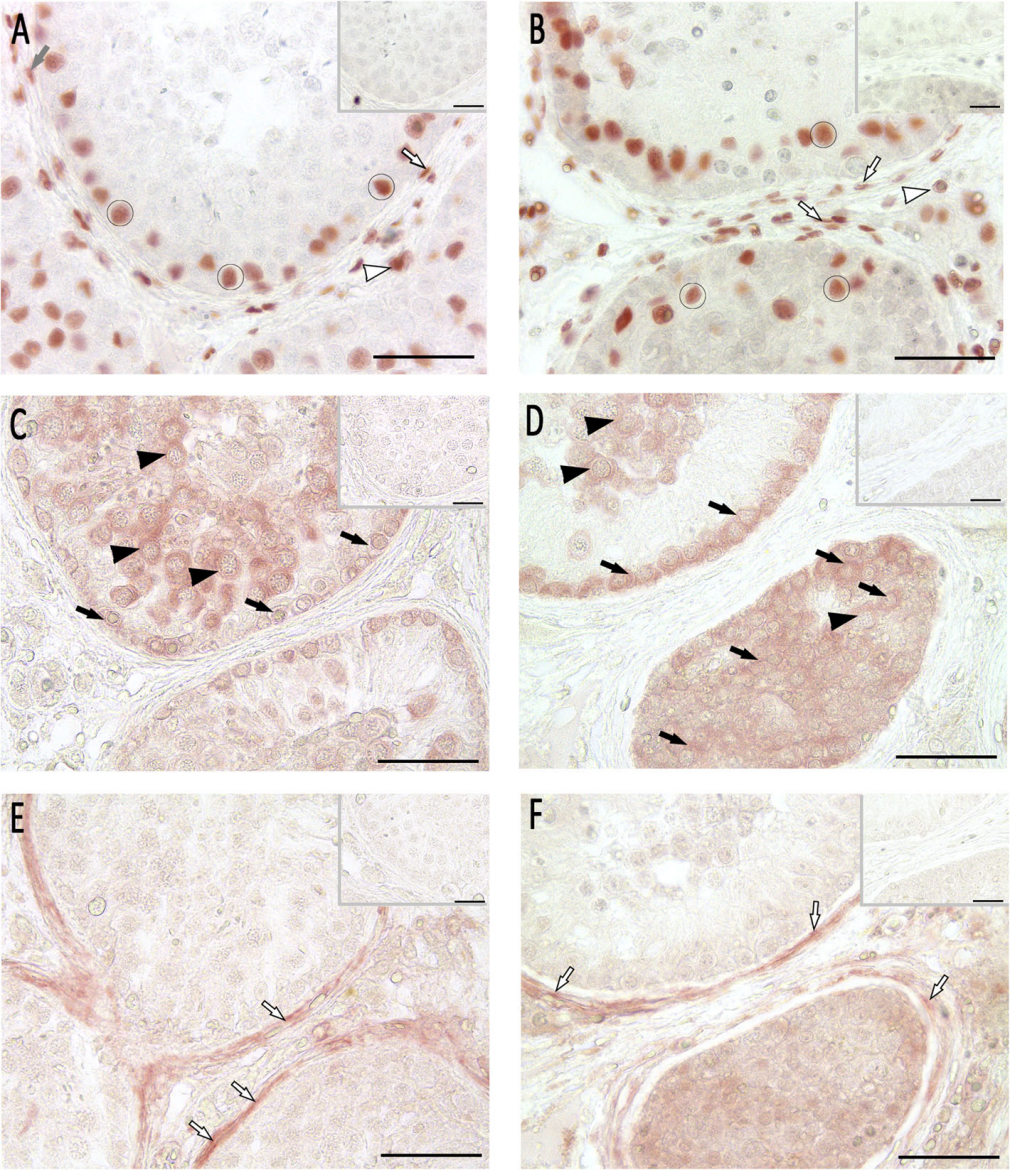
Immunohistochemical analysis of cell sex hormone receptors. Pictures **a**, **c**, and **e** show intact spermatogenesis (NSP) as control, pictures **b**, **d**, and **f** show spermatogonial arrest (patient’s left testis). **a**, **b** In both, control and spermatogonial arrest, androgen receptor (AR) was expressed in Sertoli cells (circle), peritubular myoid cells (lined arrows) and Leydig cells (lined arrowheads). **c**, **d** In both specimen, estrogen receptor alpha (ERalpha) was expressed in primary spermatocytes (black arrowhead) and spermatogonia (black arrows). **e**, **f** In NSP and spermatogonial arrest, membrane-bound G-protein coupled estrogen receptor 1 (GPER1) was expressed in peritubular myoid cells (lined arrows). AEC detection, bar 50 μm (inset bar 25 μm), inset as negative control without first antibody

In order to compare the patient’s histopathology with normal spermatogenesis (NSP), a biopsy obtained from a patient with obstructive azoospermia served as control (see above). As positive control for CK18, immunohistochemistry on testicular tissue from a patient with germ cell neoplasia in situ (GCNIS) was performed (Suppl. Fig. 3). As shown by Donner et al. [15], dysfunctional Sertoli cells in GCNIS express CK18. By using melanoma associated antigen 4 (MAGEA4) as a germ cell marker for type A and B spermatogonia, spermatogonia were detected in NSP lining up the basal membrane (Fig. 2a) and the abundant cells inside the peculiar seminiferous tubules of the patient’s left testis could be identified as spermatogonia, too (Fig. 2b). To compare the number of MAGEA4-positive spermatogonia in patient and control specimens, a semi-quantitative evaluation as outlined above was applied (for methods, see Suppl. Material, Suppl. Fig. 4) [16, 17]. Compared to NSP, the number of MAGEA4 positive cells was significantly increased in the patient’s tubules containing only spermatogonia, whereas patient’s tubules containing spermatogonia and single primary spermatocytes did not differ from NSP (Suppl. Fig. 4). Moreover, by applying distinct markers for Sertoli cells (i.e. vimentin, Fig. 2c / d, and cytokeratin 18 (CK18), Fig. 2e / f), we were able to show that Sertoli cells in both control and patient were normal in regard to intermediate filament presence and location. Comparison of NSP and patient tissue sections did not reveal marked differences in Sertoli cell staining intensity, with positive results for vimentin in all samples. In those tubules with excessive numbers of spermatogonia, distribution of vimentin was less (Fig. 2d). Staining for CK18 remained negative throughout all specimens. Staining of sex hormone receptors, such as androgen receptor (AR, Fig. 3a / b), estrogen receptor alpha (Fig. 3c / d), and membrane-bound G-protein coupled estrogen receptor 1 (GPER1, Fig. 3e / f) was comparable in the control as well as in patient samples displaying spermatogonial arrest, including those tubules with abundance of spermatogonia.

**Fig. 4:**
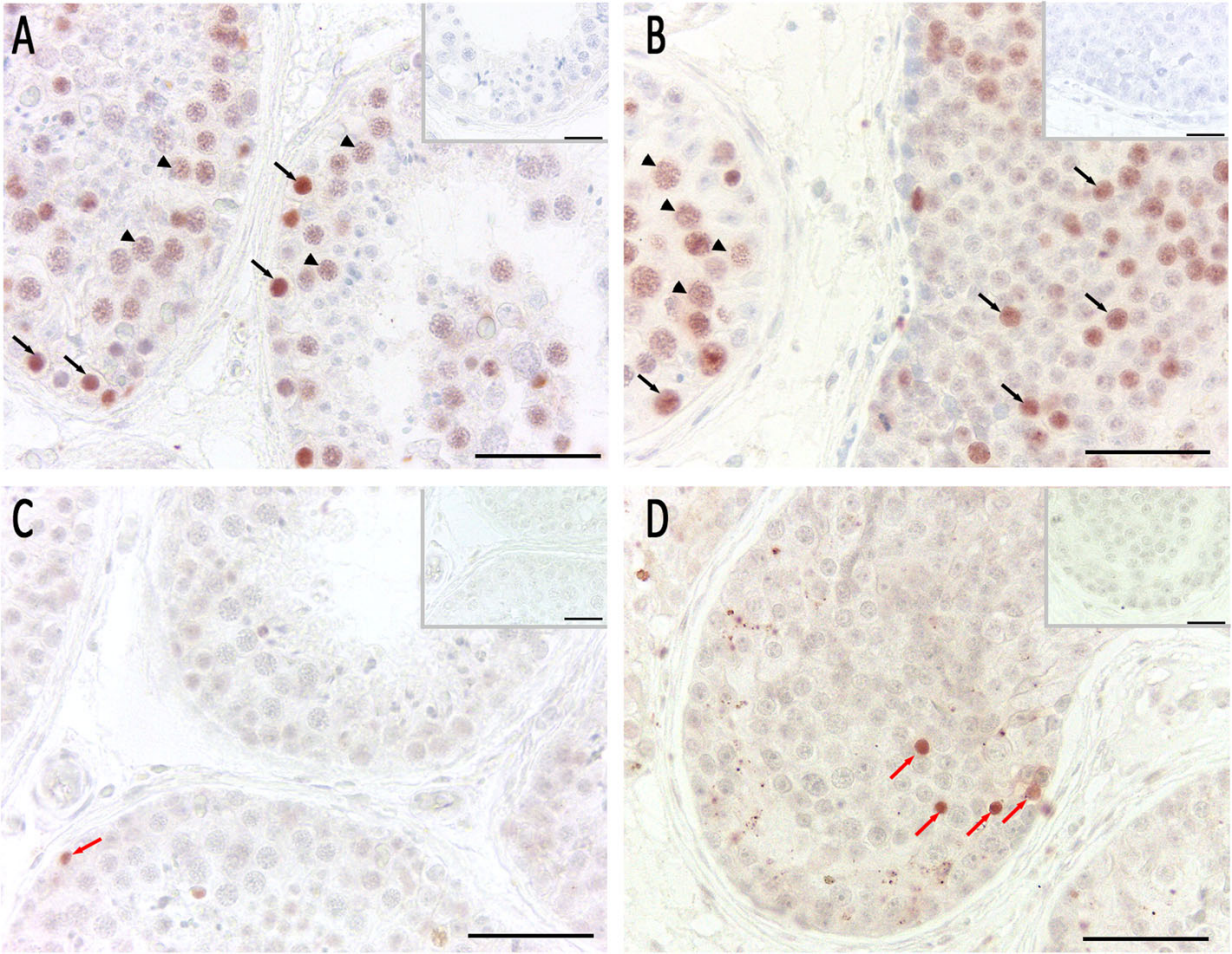
Immunohistochemical analysis of cell proliferation (**a**, **b**) and cell death (**c**, **d**). Pictures **a** and **c** show intact spermatogenesis (NSP) as control, pictures **b** and **d** show spermatogonial arrest (patient’s left testis). **a**, **b** Proliferation was tested by PCNA immunostaining and revealed intensely stained spermatogonia (black arrows) and primary spermatocytes (black arrowheads). In the spermatogonial arrest tubules, PCNA staining was detected in more spermatogonia compared to NSP. **c**, **d** TUNEL staining revealed apoptotic germ cells in NSP (red arrows) and to a higher extend in tubules with abundant spermatogonia. AEC detection, bar 50 μm (inset bar 25 μm), inset as negative control without first antibody

Proliferating cell nuclear antigen (PCNA) immunohistochemistry and Terminal deoxynucleotidyl transferase dUTP nick end labeling (TUNEL) staining were used to evaluate spermatogonial proliferation and cell death. By this, we were able to show that proliferation rate seems to be higher in tubules of the patient’s left testis revealing abundance of spermatogonia (Fig. 4a / b) compared to control. To compare the number of PCNA-positive spermatogonia in patient and control samples, the semi-quantitative evaluation outlined above was applied (for methods, see Suppl. Material, Suppl. Fig. 5) [18]. A significantly higher number of PCNA-positive spermatogonia was observed in the patient’s tubules packed with spermatogonia only, when compared to tubules with spermatogonia and single primary spermatocytes as well as NSP (Suppl. Fig. 5). DNA fragmentation as shown with TUNEL staining was also considerably higher in specimens of the patient’s left testis compared to the control (Fig. 4c / d). Again, a semi-quantitative evaluation by counting TUNEL-positive spermatogonia was applied (see above; Suppl. Fig. 6). A significantly higher number of TUNEL-positive spermatogonia could be identified in the patient’s tubules containing only spermatogonia compared to NSP (Suppl. Fig. 6).

**Fig. 5:**
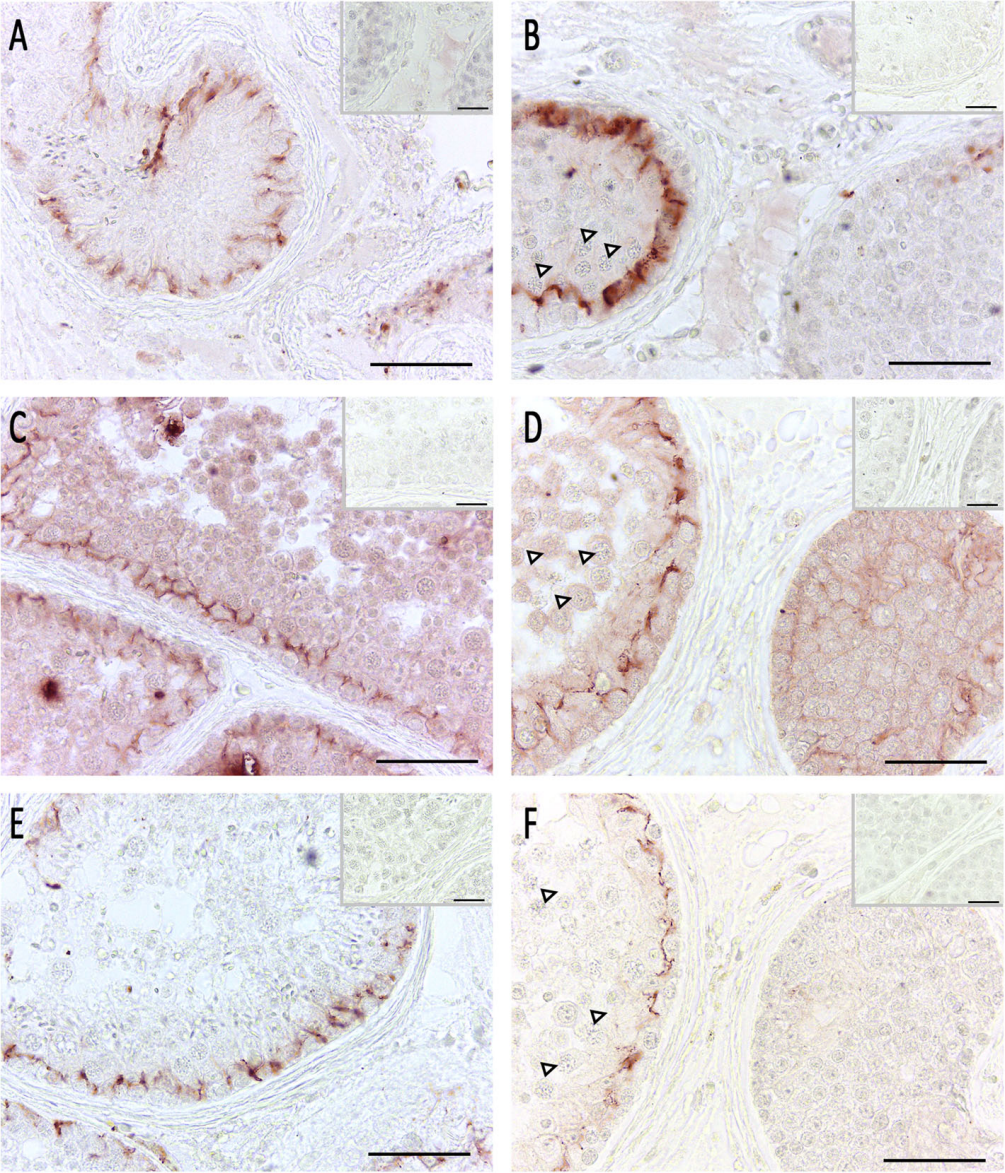
Immunohistochemical analysis of blood-testis barrier proteins. Pictures **a**, **c**, and **e** show intact spermatogenesis (NSP) as control, pictures **b**, **d**, and **f** show spermatogonial arrest (patient’s left testis). **a**, **b** ZO-1 staining in NSP (**a**) revealed a clear cytoplasmic Sertoli cell staining. In contrast, in the sample showing spermatogonial arrest (**b**), ZO-1 staining was absent in a tubule showing spermatogonia alone (right tubule), but was present in an adjacent tubule with single remaining primary spermatocytes (lined arrowheads). **c**, **d** The same staining behavior was identified for claudin-11, where a regular staining was detectable in NSP (**c**) and in single tubules with remaining primary spermatocytes (lined arrowheads) (**d**, left tubule). But no staining was detected in tubules with spermatogonia only (**d**, right tubule). **e**, **f** A similar staining pattern was identified for CX43 staining in NSP (**e**) and in single tubules with remaining primary spermatocytes (lined arrowheads) (**f**, left tubule). No staining was detected in tubules with spermatogonia only (**f**, right tubule). AEC detection, bar 50 μm (inset bar 25 μm), inset as negative control without first antibody

Sertoli cell function and blood-testis barrier formation was analyzed by ZO-1, claudin 11 and connexin (CX) 43 immunohistochemistry in control (Fig. 5a, c, and e) and spermatogonial arrest in the patient’s left testis (Fig. 5b, d, and f). Staining results indicate that these proteins were absent in tubules containing spermatogonia as the only germ cell population, or - if present - were dislocated in comparison to the control. Interestingly, in areas where single primary spermatocytes were detectable in seminiferous tubules, ZO-1, claudin 11 and CX43 were present and located correctly.

## Discussion

To our knowledge this is the first observation of an excessive, non-malignant proliferation of spermatogonia in an infertile man with NOA. Although discordant histological patterns between organs occur in up to one third of patients undergoing testicular biopsy [for review see [5], the unilateral manifestation of this unusual histopathology without any clinical signs is striking. The heterogeneity of tubular damage (“mixed atrophy”) [for review see [6] in both testes, with a spermatogenic arrest at the level of primary spermatocytes or spermatogonia as leading phenotype, argues against a common genetic background, such as testis-expressed 11 (*TEX11*) gene mutations [19]. The massively increased numbers of spermatogonia-like cells in seminiferous tubules of the left testis gives rise to the compelling question of potential malignancy. Cellular morphology, however, did not meet any criteria for germ cell neoplasia in situ (GCNIS), and immunohistochemical staining for placental alkaline phosphatase (PlAP) as a marker of atypical gonocytes proved to be negative [15, 20]. The expression of MAGEA4 is in-line with the presence of normal spermatogonia [21], though with increased proliferative activity, as reflected by immunohistochemical analysis of PCNA [22]. A clonal proliferation of MAGEA4-positive spermatogonia has been described in testes of elderly men, which may result in formation of intratubular spermatocytic seminoma in conjunction with additional mutational events [23]. However, the gross pathomorphology and cellular characteristics of this rare entity, now coined as “spermatocytic tumor”, completely differs from the histopathology seen in the left testis of our patient [24]. The moderately elevated rate of apoptotic spermatogonia (positive TUNEL assay) can be attributed to the excessive proliferation [for review see [25]. As recently reviewed by Majtnerová and Roušar [26], TUNEL assay still is regarded as one of the standard and most sensitive methods to detect apoptotic cells in tissues. As a disadvantage, TUNEL assay cannot differentiate between apoptosis, necrosis and autolytic cell death [27].

Dislocation of spermatogonia from the basement membrane has been described in testes of infertile men and was associated with disruption of the blood-testis barrier (BTB) [28]. Both, unusual proliferation of spermatogonia and absent or dislocated protein components of the BTB hint at an impairment of Sertoli cell function in the affected tubules of the patient’s left testis. Immunohistochemical analysis of different Sertoli cell markers, i.e. AR, vimentin, and CK18, confirmed the presence of mature Sertoli cells in the patient’s tissue specimens including seminiferous tubules packed with spermatogonia. AR has been described to be expressed in Sertoli cells, Leydig cells, peritubular myoid cells, and endothelial cells [29], whereas AR expression in germ cells are controversially discussed [for review see [30]. In contrast to vimentin, staining for CK18 remained entirely negative. CK18 expression characterizes immature Sertoli cells [31], and has been associated with disturbed differentiation and function of Sertoli cells in impaired spermatogenesis, focal testicular inflammation, and GCNIS [15, 30–32].

To further delineate Sertoli cell function, analysis of BTB components is mandatory [31]. One of the most commonly used techniques to assess BTB integrity are lanthanum tracer experiments [28, 32, 33], which require specialized tissue processing other than fixation with Bouin’s solution. Instead, we employed immunohistochemistry for distinct proteins of the BTB. Amongst others, ZO-1, claudin 11, and CX43 are structural components of the inter Sertoli cell junctions, which undergo alterations in conjunction with impairment of spermatogenesis [32, 34–37]. As shown by Rode et al. [38], loss of CX43 in mouse Sertoli cells leads to spermatogenic arrest at the level of spermatogonia with only single primary spermatocytes. This pattern is comparable with the patient’s peculiar testicular histopathology reported here. Of note, BTB components included in the immunohistochemical analysis were absent and/or dislocated in tubules containing only spermatogonia. These observations provide suggestive evidence that BTB integrity and related Sertoli cell function is impaired.

Moreover, restoration of spermatogenesis, e.g. after heat-induced damage, strongly depends on Sertoli cells, BTB formation and other Sertoli cell-derived factors such as glial cell line-derived neurotrophic factor (GDNF) (for review see [39]). In mice overexpressing GDNF, high numbers of undifferentiated, type A-like spermatogonia were populating the testicular cords [40].

A key question of concern is whether CC treatment of the patient over more than 6 months has either induced or promoted excessive increase in numbers of spermatogonia in seminiferous tubules of the left testis. Apart from the enhancement of gonadotrophin levels via blockage of the negative feedback of estrogens at the level of the hypothalamus and the pituitary, and thus, indirect action on testicular function, direct effects of CC on seminiferous tubules should also be considered. As a pre-requisite, expression of estrogen receptor (ER) alpha and beta (ERbeta) was shown in human spermatogonia [17]. ERbeta and G-protein coupled membrane estrogen receptor (GPER) could be detected in testicular somatic cells, namely Sertoli cells [17]. In accordance with these data, positive staining for ERalpha was found in patient’s tubules, including those with excessive proliferation of spermatogonia, whereas GPER-1 was localized in peritubular cells. Wagner et al. [41] reported that proliferation of ERalpha-positive spermatogonia could be partially restored by administration of estradiol in GnRH immune-castrated boars. On the other hand, administration of CC delayed BTB formation in rats and lead to a significantly decreased expression of CX43 in granulosa cells of baboons [42, 43]. In conclusion, both intrinsic estrogen-agonistic and antagonistic properties of CC [44] have to be considered as driving forces underlying the unusual pathology seen in our patient. Considering that heterogeneity of testicular damage, even within individual organs, is common (for review see [5]), the unilateral manifestation of tubules with excessive increase in numbers of spermatogonia is plausible. As a limitation of our study, tissue specimens of preceding TESE attempts, i.e. those before CC treatment, were not available for longitudinal histopathological evaluation.

Although CC dosages of 25 to 50 mg per day are well tolerated by the majority of patients [45], the risk of adverse effects of should not be neglected. Early observational studies in healthy volunteers and oligozoospermic patients revealed both non-responders and cases with deterioration of semen quality under CC therapy [46]. Notably, Pasqualotto et al. [47] reported three patients with severe oligozoospermia becoming azoospermic after CC treatment.

## Conclusion

The striking testicular histopathology with excessive, non-malignant proliferation of spermatogonia seen in our patient draws attention to possible mechanisms how CC could exert negative effects on spermatogonia and/or Sertoli cells, along with BTB integrity and function. Although underlying molecular details remain to be elucidated, we hypothesize that the unusual pathology of seminiferous tubules was triggered by the extensive and high-dose clomiphene citrate treatment preceding testicular biopsy. Whether CC exerts direct effects, or pre-existent tubular damage with concomitant disturbance of BTB facilitates testicular adverse reactions may be a matter of debate. Thus, the case report illustrates the need for randomized clinical trials investigating anti-estrogens and other compounds used off-label for improvement of TESE success.

## Supplementary information

**Additional file 1.** Supplementary material [6, 16–18, 48]

## Data Availability

All data generated or analysed during this study are included in this published article [and its supplementary information files].

## Abbreviations

A. dest: Distilled water
AEC: Aminoethyl carbazole
AR: Androgen receptor
BSA: Bovine serum albumin
BTB: Blood-testis barrier
CC: Clomiphene citrate
CFTR: Cystic fibrosis transmembrane conductance regulator
CK18: Cytokeratin 18
Cldn11: Claudin 11
cTESE: conventional testicular sperm extraction
CX43: Connexin 43
DAB: 3,3′-diaminobenzidine
EDTA: Ethylenediaminetetraacetic acid
ERalpha: Estrogen receptor alpha
ERbeta: Estrogen receptor beta
FSH: Follicle stimulating hormone
GCNIS: Germ cell neoplasia in situ
GDNF: Glial derived neurotrophic factor
GPER1: G-protein coupled estrogen receptor 1
HE: Hematoxylin eosin
HZRM: Hessian Center for Reproductive Medicine
ICSI: Intracytoplasmic sperm injection
IU/l: International unit per liter
IVF: In vitro fertilization
LH: Luteinizing hormone
MAGEA4: Melanoma associated antigen 4
mTESE: microscopically assisted testicular sperm extraction ng/ml (nanogram per milliliter)
nmol/l: nanomole per liter
NOA: Non-obstructive azoospermia
NSP: Normal spermatogenesis
PBS: Phosphate buffered saline
PCNA: Proliferating cell nuclear antigen
pg/ml: picogram per milliliter
PlAP: Placental alkaline phosphatase
SD: Standard deviation
SHBG: Sex hormone binding globulin
SGA: Spermatogonia arrest, tubules with solely spermatogonia
SZA: Spermatocyte arrest, tubules with spermatogonia and primary spermatocytes
TESE: Testicular sperm extraction
TEX11: Testis-expressed 11
TUNEL: Terminal deoxynucleotidyl transferase dUTP nick end labelling
ZO-1: Zona occludens protein 1
