# Supplementary material for: Excessive unilateral proliferation of spermatogonia in a patient with non-obstructive azoospermia – adverse effect of clomiphene citrate pre-treatment?
Source: Basic Clin Androl. 2020 Sep 1;30:13. doi: 10.1186/s12610-020-00111-7 (PMC7461256; doi:10.1186/s12610-020-00111-7)
Supplement: Supplementary file 1 — Additional file 1. Supplementary material [6, 16–18, 48] [file 12610_2020_111_MOESM1_ESM.zip › Suppl Material.docx]

**Supplementary Material**

Histological evaluation of testicular biopsies

Processing of testicular tissue, histological evaluation and score count analysis of spermatogenesis were performed as previously described [48]. Testicular tissue was fixed in Bouin’s solution for at least five hours, dehydrated in ascending alcohol series and embedded in paraffin wax. Subsequently, sections of 5µm thickness were cut on a microtome, and mounted on coated glass slides. After drying of sections overnight in a 36°C incubator, sections were dewaxed and rehydrated in descending alcohol series and stained using hematoxylin and eosin according to standard protocols. Using score count analysis, percentage of tubules containing elongated spermatids (with score count = 10 meaning 100% of tubules contain elongated spermatids) can be calculated (Suppl. Fig. 1; [6]).

TUNEL assay

To assess DNA fragmentation and therefore apoptosis in the biopsies, we performed a semi-quantitative TUNEL (terminal desoxynucleotidyl transferase dUTP nick and labeling) assay as described earlier [18]. For this, we used the ApopTagH Peroxidase In Situ Apoptosis Detection Kit (Millipore, MA, USA) as recommended by the manufacturer. Briefly, sections were dewaxed in a descending alcohol series and cooked in citrate buffer (pH = 6) in a common microwave oven for 15 min. Endogenous peroxidase was blocked by incubation in 3% H_2_O_2_ for 5 min and sections were washed subsequently twice for 5 min each in PBS. Equilibration buffer was applied undiluted. These steps were performed at room temperature. Subsequently, sections were incubated with TdT enzyme (1:20, 20µl per section) for 1 h at 37°C. Afterwards, the sections were left in Stop/Washing Buffer solution overnight. The next day, sections were washes three times for 1 min each in phosphate buffered saline (PBS) and incubated with anti-digoxigenin-conjugate (undiluted, 20 µl per section) for 30 min at room temperature. Sections were washed again four times for 2 min each in PBS and staining was visualized by 3,3′-diaminobenzidine (DAB) incubation for 5 min at room temperature. Sections were rinsed in distilled water (A. dest) three times for 5 min and counterstained with Meyer’s hematoxylin. After ascending alcohol series, sections were mounted in Eukitt.

Immunohistochemistry

Using various antibodies, the identity of cells, their proliferative activity, and the degree of maturation was analyzed. For this purpose, immunohistochemistry was performed as described previously [16, 17]. Shortly, 5µm thick sections were cut, dewaxed and rehydrated. For antigen retrieval, sections were boiled in a citrate buffer solution at pH 6 (MAGEA4, PCNA, ZO-1, Cx43, vimentin, ERalpha, GPER1), ethylenediaminetetraacetic acid (EDTA) at pH11 (claudin 11, AR) or proteinase K (10 µg/ml, CK18) in a common microwave oven or at 37°C for 15 min, respectively. Endogenous peroxidases were blocked by incubation of sections in 3% H_2_O_2_ solution in Tris buffer for 30 min. After treatment with 5% bovine serum albumin (BSA) in Tris buffer for 30 min to block unspecific binding sites, primary antibodies were applied and incubated at 4°C overnight. The next day, sections were washed and secondary antibodies were applied for 30 min at room temperature. Primary and secondary antibodies and their dilutions can be seen in Suppl. Tab. 2. Immunoreactions were visualized using peroxidase conjugated streptavidin (Vectastain Elite ABC standard Kit Peroxidase, Vector Laboratories, Burlingame, CA, USA) for 30 min at room temperature followed by AEC staining (Biologo, Kronshagen, Germany). For negative control, primary antibodies were replaced by Tris buffer. Sections were mounted with Kaiser’s glycerol gelatin (Merck, Darmstadt, Germany).

Immunohistochemistry was performed on NSP as internal positive control and a representative section from the left testis (lower pole).

Semi-quantitative analysis of histological and immunohistochemical sections

To compare numbers of spermatogonia in histology and immunohistochemistry between the control and the patient, we performed a semi-quantitative analysis.

For this approach, spermatogonia were counted in 25 (if available) randomly chosen tubules from three categories: 1) tubules from the control’s testis, 2) tubules from the patient’s testis with an arrest at the level of primary spermatocytes (i.e. containing spermatogonia and primary spermatocytes) and 3) tubules from the patient’s testis with an arrest at the level of spermatogonia (i.e. containing only spermatogonia). Especially for the second category, the number of tubules available for analysis was lower than 25, as this manifestation was rare in the patient’s tissue. For analysis of HE stains of the third category, only 10 tubules were included due to the extremely high number of spermatogonia. Semi-quantitative analysis was performed on sections after HE staining (Suppl. Fig. 2), MAGEA4 staining (Suppl. Fig. 4), PCNA staining (Suppl. Fig. 5), and TUNEL assay (Suppl. Fig. 6). For statistical evaluation, we applied a Tukey’s multiple comparison test after One way ANOVA (GraphPad Prism 7).

**Suppl. Table 1 Semen analysis of the patient**

| **Parameter** | **05/2018^#^** |
| --- | --- |
| Sexual abstinence (days) | 3 |
| Volume (ml) | 2,2 |
| Consistency | normal |
| pH | 7,4 |
| Sperm concentration [Mio/ml] | 0 |
| after centrifugation | 0 |
| Leukocytes (peroxidase positive cells) [Mio./ml] | 0,1 |
| PMN Elastase [ng/ml] | 18 |
| Fructose [µmol/Ej] | 16,4 |
| α-Glukosidase [mU/Ej] | 18,1 |
| Semen culture / STI-PCR | negative |

# after clomiphene citrate treatment, prior to 4^th^ testicular biopsy / TESE (see Table 1)

ml milliliter, Mio/ml million sperm per milliliter, ng/ml nanogram per milliliter, µmol/Ej micromole per ejaculate, mU/Ej milliunits per ejaculate

| **Primary Antibody** | **Dilution** | **Secondary Antibody** | **Dilution** |
| --- | --- | --- | --- |
| PCNA  monoclonal mouse anti-human  NCL-L-PCNA, LeicaBiosystems | 1:100 | Peroxidase-conjugated AffiniPure Goat Anti-Mouse IgG, #115-035-003, Dianova | 1:400 |
| CK18  monoclonal mouse anti-human, M7010, Dako | 1:50 |  | 1:400 |
| Vimentin  polyclonal rabbit anti-human, sc-7557, Santa Cruz | 1:2000 | Peroxidase-conjugated AffiniPure Goat Anti-Rabbit IgG, #111-035-003, Dianova | 1:100 |
| Cldn11  polyclonal rabbit anti-human, ab27565, Abcam | Prediluted |  | 1:400 |
| ZO-1  polyclonal rabbit anti-human, #61-7300, Invitrogen | 1:250 |  | 1:400 |
| Cx43  polyclonal rabbit anti-human, #3512, Cell Signaling | 1:50 |  | 1:400 |
| AR  monoclonal rabbit anti-human, LS-B7258/55408, LSBio | 1:50 |  | 1:200 |
| ERalpha (HC-20)  Polyclonal rabbit anti-human,  Sc-543, Santa Cruz | 1:100 |  | 1:200 |
| GPER1  polyclonal rabbit anti-human,  LS-A4271, Biozol | 1:100 |  | 1:200 |
| MAGEA4  polyclonal rabbit anti-human, HPA021942, Atlas Antibodies | 1:2000 |  | 1:400 |

**Suppl. Table 2 Primary and secondary antibodies used in this study**

PCNA: proliferating cell nuclear antigen, CK18: cytokeratin 18, Cldn11: claudin 11, ZO-1: zona occludens protein 1, Cx43: connexin 43, AR: androgen receptor, ERalpha: estrogen receptor alpha, GPER: G-protein coupled estrogen receptor 1, MAGEA4: melanoma associated antigen 4

**Suppl. Fig. 1 Testicular biopsy evaluation sheet [for review see [6]**

**Suppl. Fig. 2 Semi-quantitative analysis of number of spermatogonia in histological staining**

Spermatogonia were counted in seminiferous tubules from a testis with normal spermatogenesis (NSP, n = 25, white bar), tubules of the patient’s left testis containing only spermatogonia (SGA, n = 10, light gray bar) or spermatogonia and some primary spermatocytes (SZA, n = 7, dark gray bar). Counts are depicted as means and standard deviation (SD). *** p = <0.001

**Suppl. Fig. 3 Positive control cytokeratin 18 immunohistochemistry**

**A** Immunohistochemistry for cytokeratin 18 (CK18) in a patient showing germ cell neoplasia in situ (GCNIS). GCNIS cells are indicated by arrowheads, CK18 positive Sertoli cell cytoplasm is indicated by black arrows.

**B** Negative control. Exemplary GCNIS cells are again indicated by arrowheads.

AEC detection, bar 50µm, hematoxylin counterstain. Primary magnification x40.

**Suppl. Fig. 4 Semi-quantitative analysis of number of spermatogonia after MAGEA4 immunohistochemical staining**

Spermatogonia were counted in seminiferous tubules from a testis with normal spermatogenesis (NSP, n = 25, white bar), tubules of the patient’s left testis containing only spermatogonia (SGA, n = 25, light gray bar) or spermatogonia and some primary spermatocytes (SZA, n = 10, dark gray bar). Counts are depicted as means and standard deviation (SD). *** p = <0.001

**Suppl. Fig. 5 Semi-quantitative analysis of number of spermatogonia after PCNA immunohistochemical staining**

Spermatogonia were counted in seminiferous tubules from a testis with normal spermatogenesis (NSP, n = 25, white bar), tubules of the patient’s left testis containing only spermatogonia (SGA, n = 25, light gray bar) or spermatogonia and some primary spermatocytes (SZA, n = 4, dark gray bar). Counts are depicted as means and standard deviation (SD). *** p = <0.001

**Suppl. Fig. 6 Semi-quantitative analysis of number of spermatogonia after TUNEL staining**

Spermatogonia were counted in seminiferous tubules from a testis with normal spermatogenesis (NSP, n = 25, white bar), tubules of the patient’s left testis containing only spermatogonia (SGA, n = 25, light gray bar) or spermatogonia and some primary spermatocytes (SZA, n = 7, dark gray bar). Counts are depicted as means and standard deviation (SD). * p = <0.05
