# Supplementary material for: Excessive unilateral proliferation of spermatogonia in a patient with non-obstructive azoospermia – adverse effect of clomiphene citrate pre-treatment?
Source: Basic Clin Androl. 2020 Sep 1;30:13. doi: 10.1186/s12610-020-00111-7 (PMC7461256; doi:10.1186/s12610-020-00111-7)
Supplement: Supplementary file 1 — Additional file 1. Supplementary material [6, 16–18, 48] [file 12610_2020_111_MOESM1_ESM.zip › Suppl. Fig. 1.pdf]

# Evaluation of testicular biopsy

**Name, prename:** **Date of birth:**

**Date of biopsy:**

**Clinic:**

**Clinical diagnosis:** normogonadotropic azoospermia

| **criteria** | **Biopsy right** | | | | **Biopsy left** | | | |
|---|---|---|---|---|---|---|---|---|
| **semiquantitative** | **Number of tubules** | | | | **Number of tubules** | | | |
| **Spermatogenesis up to** | **1** | **2** | **3** | **4** | **5** | **6** | **7** | **8** |
| elongated spermatids | 1 | 1 | 1 | | | | | |
| round spermatids | 1 | 2 | 2 | | | | | |
| primary spermatocytes | 90 | 130 | 80 | 50 | | | 10 | |
| spermatogonia | | | | | 90 | 100 | 210 | 27 |
| Sertoli cell only | | 3 | | | | | | |
| tubular shadows | 15 | 21 | 15 | | 35 | | | |
| | | | | | | | | |
| total | 105 | 90 | 150 | 50 | 125 | 100 | 220 | 27 |
| score | 0.1 | 0.1 | 0.07 | 0 | 0 | 0 | 0 | 0 |
| | | | | | | | | |
| **Morphological** | | | | | | | | |
| tubules containing | | | | | | | | |
| multinuclear spermatids | | | | | | | | |
| multinuclear spermatocytes | | | | | | | | |
| multinuclear spermatogonia | | | | | | | | |
| megalospermatocytes | | | | | | | | |
| megalospermatogonia | | | | | | | | |
| degenerative germ cells | | +++ | all | all | | | | |
| tubular diverticle | | | | | | | | |
| thickening of lamina propria | ± | ± | ± | ± | | | | |
| morphology of Sertoli cells | | | | | | | | |
| morphology of Leydig cells | | | | | | | | |
| interstitium | | | | | | | | |
| peculiar features | | | | | massive numbers of spermatogonia-like germ cells | | | |
| diagnosis | Arrest at level of primary spermatocytes, total atrophy, hypospermatogenesis | | | | Arrest at the level of spermatogonia | | | |
