# Supplementary figures and images for: Excessive unilateral proliferation of spermatogonia in a patient with non-obstructive azoospermia – adverse effect of clomiphene citrate pre-treatment?
Source: Basic Clin Androl. 2020 Sep 1;30:13. doi: 10.1186/s12610-020-00111-7 (PMC7461256; doi:10.1186/s12610-020-00111-7)

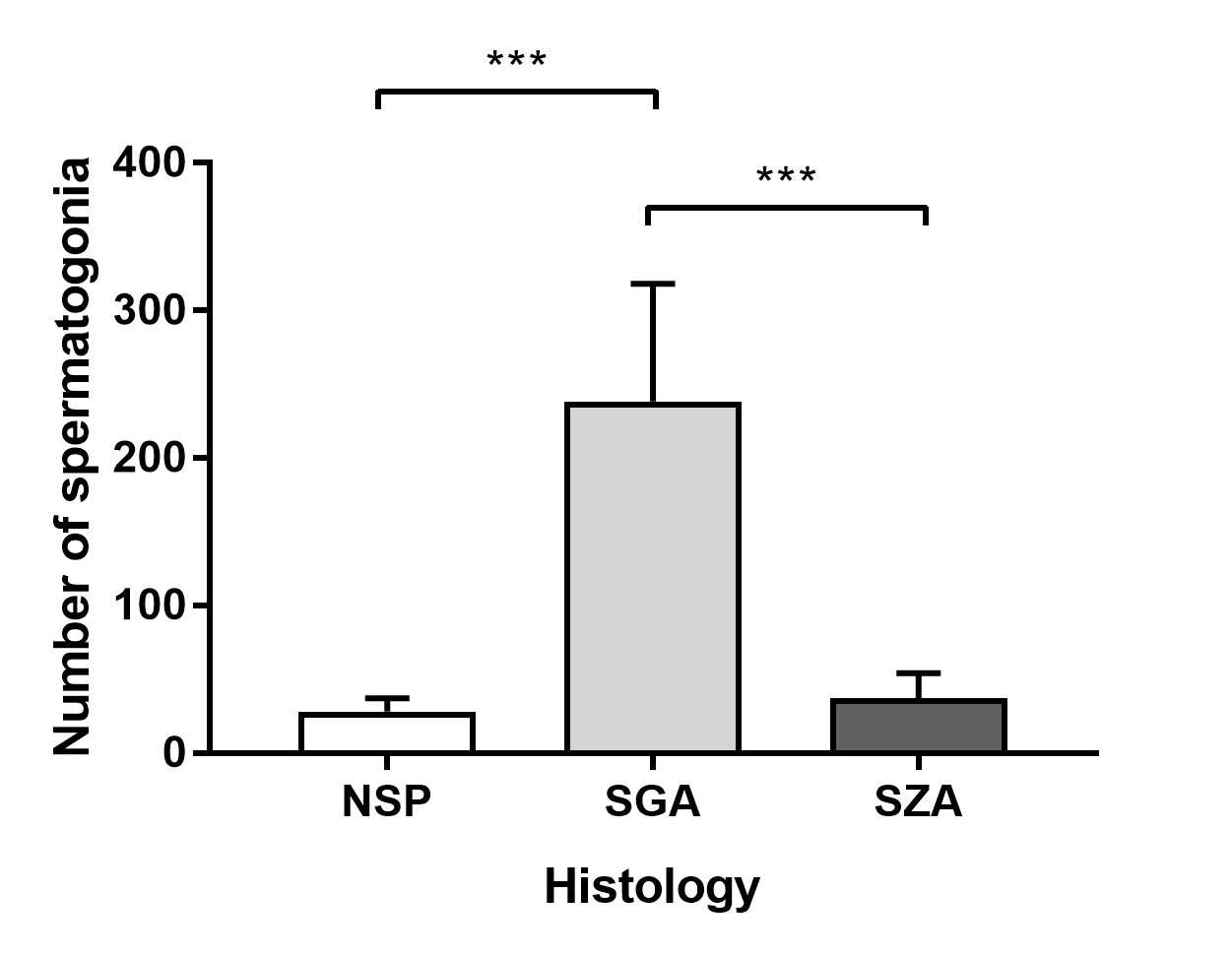

Supplement: Supplementary file 1 — Additional file 1. Supplementary material [6, 16–18, 48] [file 12610_2020_111_MOESM1_ESM.zip › Suppl. Fig. 2 (HE).tif]

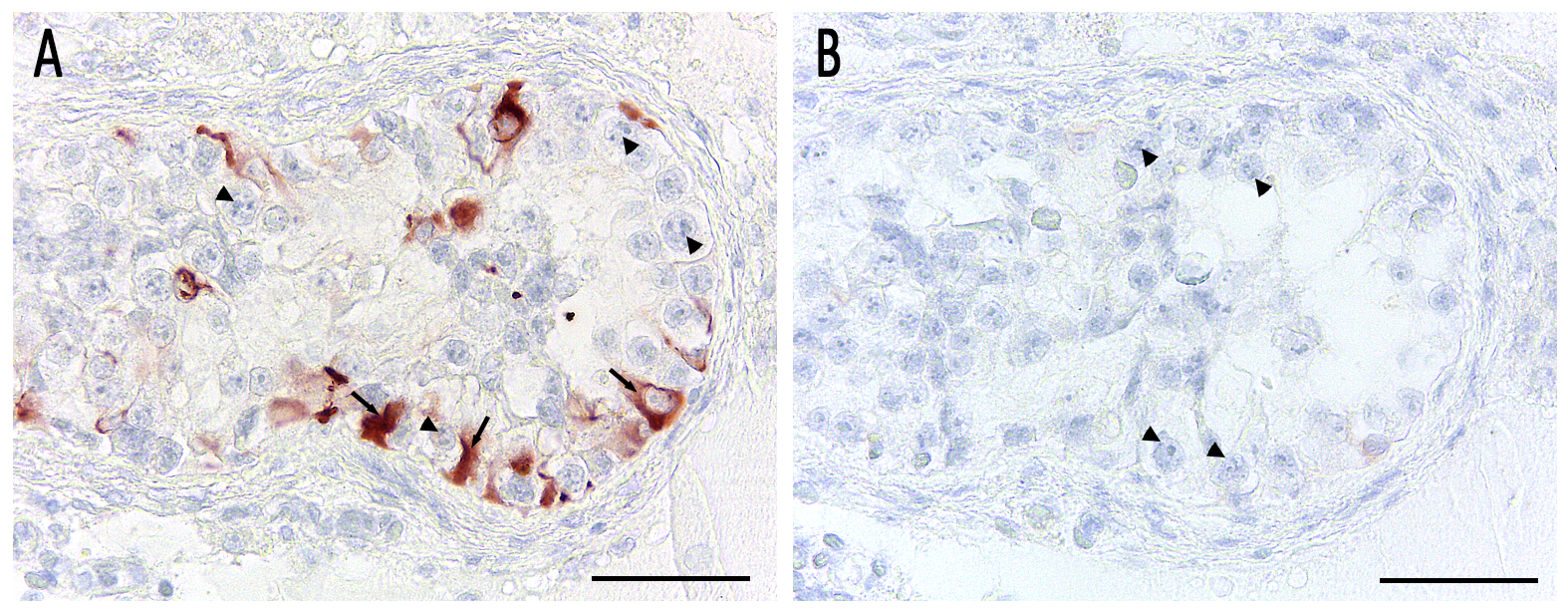

Supplement: Supplementary file 1 — Additional file 1. Supplementary material [6, 16–18, 48] [file 12610_2020_111_MOESM1_ESM.zip › Suppl. Fig. 3 (CK18).tif]

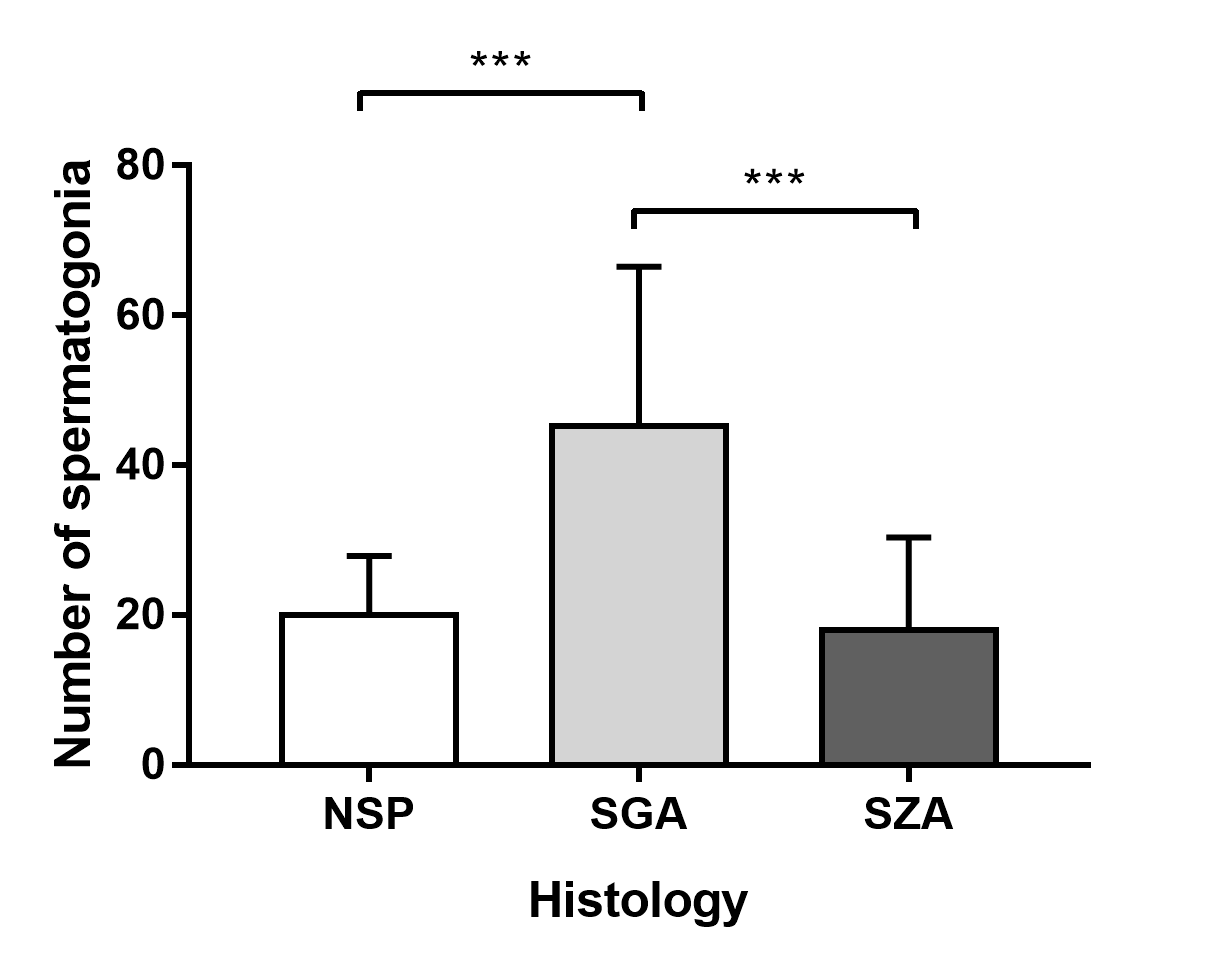

Supplement: Supplementary file 1 — Additional file 1. Supplementary material [6, 16–18, 48] [file 12610_2020_111_MOESM1_ESM.zip › Suppl. Fig. 4 (MAGEA4).tif]

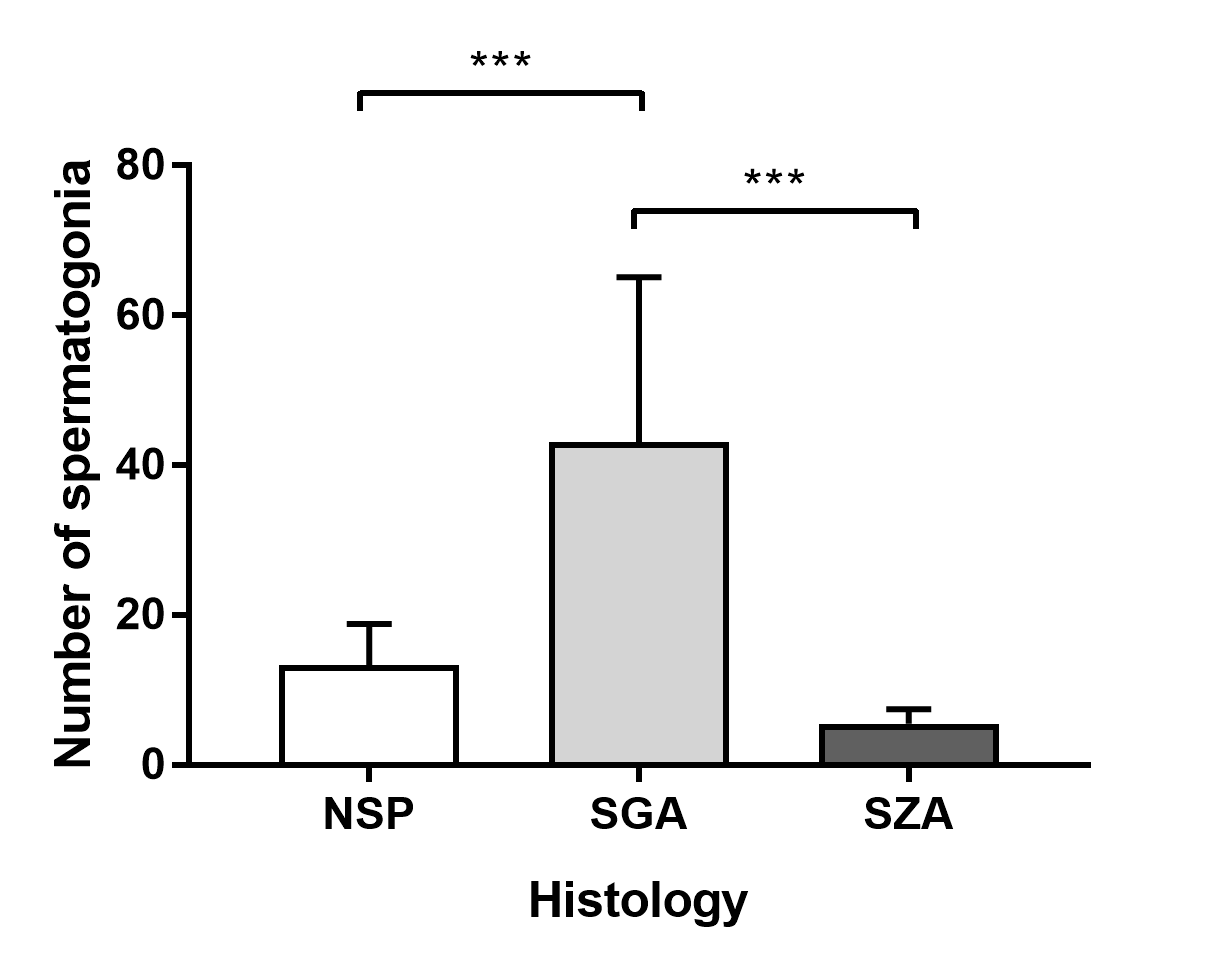

Supplement: Supplementary file 1 — Additional file 1. Supplementary material [6, 16–18, 48] [file 12610_2020_111_MOESM1_ESM.zip › Suppl. Fig. 5 (PCNA).tif]

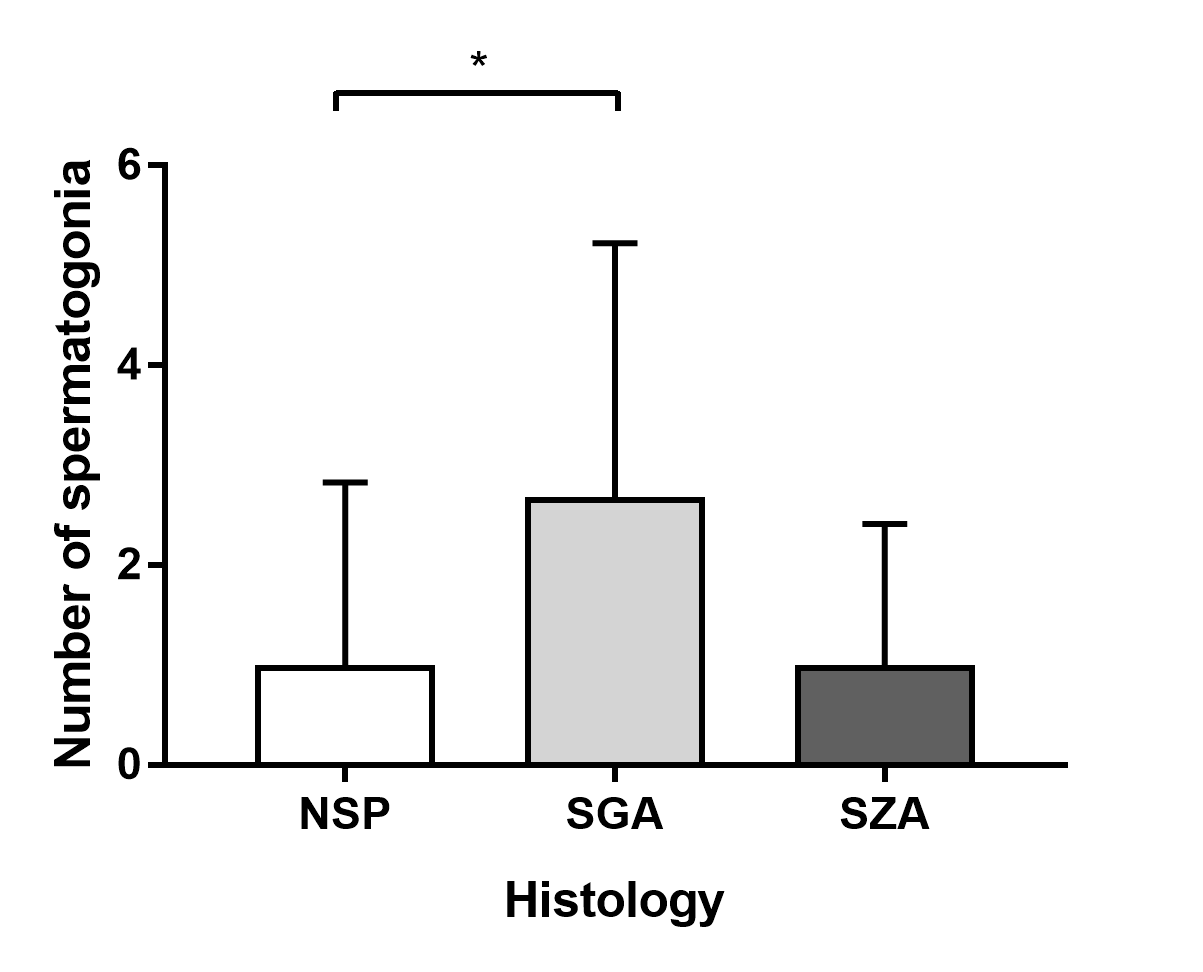

Supplement: Supplementary file 1 — Additional file 1. Supplementary material [6, 16–18, 48] [file 12610_2020_111_MOESM1_ESM.zip › Suppl. Fig. 6 (TUNEL).tif]
